# Permanent draft genome sequence of *Vibrio tubiashii* strain NCIMB 1337 (ATCC19106)

**DOI:** 10.4056/sigs.1654066

**Published:** 2011-04-29

**Authors:** Ben Temperton, Simon Thomas, Karen Tait, Helen Parry, Matt Emery, Mike Allen, John Quinn, John MacGrath, Jack Gilbert

**Affiliations:** 1 Plymouth Marine Laboratory, Prospect Place, The Hoe, Plymouth, UK; 2 Queen’s University Belfast, School of Biological Sciences, Medical Biology Centre, Belfast, Northern Ireland; 3 University of Plymouth, Department of Microbiology, Drakes Circus, Plymouth; 4 Argonne National Laboratory, Argonne, IL, USA; 5 Department of Ecology and Evolution, University of Chicago, Chicago, IL, USA

## Abstract

*Vibrio tubiashii* NCIMB 1337 is a major and increasingly prevalent pathogen of bivalve mollusks, and shares a close phylogenetic relationship with both *V. orientalis* and *V. coralliilyticus*. It is a Gram-negative, curved rod-shaped bacterium, originally isolated from a moribund juvenile oyster, and is both oxidase and catalase positive. It is capable of growth under both aerobic and anaerobic conditions. Here we describe the features of this organism, together with the draft genome and annotation. The genome is 5,353,266 bp long, consisting of two chromosomes, and contains 4,864 protein-coding and 86 RNA genes.

## Introduction

The genus *Vibrio* is both numerous and ubiquitous within marine environments, with *Vibrio* species harbored within many diverse marine organisms, such as mollusks, shrimps, fishes, cephalopods and corals [1]. Comparative genome analysis has revealed a huge genetic diversity within this genus, which is driven by mutations, chromosomal rearrangements, loss of genes by decay or deletion, and gene acquisitions through duplication or horizontal transfer (e.g. the acquisition of bacteriophages, pathogenicity islands, and super-integrons), the combination of which presumably stimulates genetic and functional diversity and allows this group to colonize a wide variety of ecological niches and hosts [1,2].

*Vibrio tubiashii* was first described as three strains of *Vibrio anguillarum* by Tubiash *et al* [3] in 1965. The organisms were isolated from bivalve mollusks during an outbreak of bacillary necrosis in Milford, Connecticut, and deposited in the American Type Culture Collection as ATCC 19105, 19106 and 19109. These three strains were further elucidated and formally named as *V. tubiashii* by Hada *et al* [4] in 1984. Subsequently, several virulence factors have been identified [5,6] and the organism is increasingly implicated in major disease outbreaks in bivalve mollusks [1].

*V. tubiashii* is closely related to the proposed coral pathogen *V. coralliilyticus,* as well as *V. orientalis*, a bacterium associated with penaeid shrimps [7]. Indeed, *V. coralliilyticus* was initially designated as a *V. tubiashii* strain [8,9] due to their close similarity.

## Classification and features

*Vibrio tubiashii* 1337 belongs to the Gammaproteobacteria and are contained within the family, *Vibrionaceae* [Table 1]. Cells of *Vibrio tubiashii* are Gram-negative curved-rods of approximately 0.5 by 1.5 µm, which are motile in liquid media by means of a single sheathed, polar flagellum [3,4] These cells are facultative anaerobes, [3,4,22]. It is catalase and oxidase positive, capable of splitting indole from tryptophan, and can use glucose, xylose, mannitol, rhamnose, sucrose, arabinose and acetate as sole carbon sources, and has β-galactosidase activity, despite an apparent inability to ferment lactose. *V. tubiashii* is capable of dissimilatory nitrate and nitrite reduction under anaerobic conditions, can use organic phosphorus during phosphate limitation, and can utilize 2-aminoethylphosphonate as a sole phosphorus source.

**Table 1:** Classification and general features of *V. tubiashii* according to the MIGS recommendations

**MIGS ID**	**Property**	**Term**	**Evidence code**
		Domain *Bacteria*	TAS [10]
		Phylum *Proteobacteria*	TAS [11]
		Class *Gammaproteobacteria*	TAS [12,13]
	Current classification	Order *Vibrionales*	TAS [14]
		Family *Vibrionaceae*	TAS [15,16]
		Genus *Vibrio*	TAS [15,17-19]
		Species *Vibrio tubiashii* NCIMB 1337	TAS [4]
	Gram stain	negative	IDA
	Cell shape	Curved rods (vibroid)	IDA
	Motility	motile via single polar flagellum	IDA
	Sporulation	Non-sporulating	IDA
	Temperature range	Mesophile 12-30^o^C	IDA
	Optimum temperature	25^o^C	IDA
MIGS 6.3	Salinity	Slightly halophylic, optimum 1-3% NaCl	IDA
MIGS-22	Oxygen requirement	Aerobic/ facultative anaerobic	IDA
	Carbon source	Highly diverse	IDA
	Energy source	Highly diverse	IDA
MIGS-6	Habitat	Marine invertebrates	TAS [20]
MIGS-16	Biotic relationship	Parasitic	TAS [3]
MIGS-14	Biosafety level	2	TAS [4]
	Isolation	Moribund juvenile oyster (*Crassostrea virginica*)	TAS [3,4]
MIGS-4	Geographical location	Milford, Connecticut, USA	TAS [3]
MIGS-5	Sample collection time	01/02/1965	TAS [3]
MIGS 4.1	latitude	41.22 N	TAS [3]
MIGS 4.2	longitude	-73.06 W	TAS [3]
MIGS 4.3	Depth	Not reported	
MIGS 4.4	Altitude	Marine	TAS [3]
			
			

Evidence codes - IDA: Inferred from Direct Assay (first time in publication); TAS: Traceable Author Statement (i.e., a direct report exists in the literature); NAS: Non-traceable Author Statement (i.e., not directly observed for the living, isolated sample, but based on a generally accepted property for the species, or anecdotal evidence). These evidence codes are from the Gene Ontology project [21]. If the evidence code is IDA, then the property was directly observed, for a live isolate by one of the authors, or an expert or reputable institution mentioned in the acknowledgements.

*V. tubiashii* has an absolute requirement for sodium and chloride ions, and is incapable of growth on media containing less than 0.5% W/V NaCl. The temperature optimum for growth is 25^o^C, but growth does occur  in the range of 12-30^o^C. The organism is killed at 37^o^C. *V. tubiashii* has a biphasic pH response and grows optimally at both pH 8.0 and 6.5, but displays weakened growth at pH 7.0 and 7.5. The bacterium shows rapid growth on marine broth and produces buff colored, opaque, irregular, slightly convex colonies on marine agar, and yellow colonies, characteristic of the *Vibrionaceae,* on Thiosulfate-Citrate-Bile-Sucrose Agar (TCBS).

### Growth conditions and DNA isolation

*Vibrio tubiashii* NCIMB 1337 (ATCC19106) was grown in marine broth (seawater + 1 gl^-1^ yeast extract and 0.5 gl^-1^ tryptone) at 25^o^C for 24 hours. DNA was extracted using the Qiagen DNAeasy blood and tissue kit, without modification of the manufacturer’s protocol.

## Genome sequencing and annotation

### Genome sequencing

The genome was sequenced using the Illumina sequencing platform. All general aspects of library construction and sequencing performed at the NERC Biomolecular analysis facility can be found on the NBAF website [23]. SOLEXA Illumina reads were assembled using VELVET Large Newbler contigs that were broken into 4,074 overlapping fragments of 1,000 bp and entered into the assembly as pseudo-reads. The sequences were assigned quality scores based on consensus q-scores with modifications to account for overlap redundancy and to adjust inflated q-scores. The error rate of the completed genome sequence is less than 1 in 100,000. Overall sequencing provided 131 × coverage of the genome.

### Genome annotation

Genes were identified using the RAST server The predicted CDSs were translated and used to search the National Center for Biotechnology Information (NCBI) nonredundant database, UniProt, TIGRFam, Pfam, PRIAM, KEGG, COG, and InterPro databases. The tRNAScanSE tool [24] was used to find tRNA genes, whereas ribosomal RNAs were found by using BLASTn against the ribosomal RNA databases. The RNA components of the protein secretion complex and the RNaseP were identified by searching the genome for the corresponding Rfam profiles using INFERNAL [25]. Additional gene prediction analysis and manual functional annotation was performed within the Integrated Microbial Genomes (IMG) platform developed by the Joint Genome Institute, Walnut Creek, CA, USA [26,27].

### Genome project information

This organism was selected for sequencing on the basis of its increasing impact as a bivalve pathogen, and was funded by *i*-G Peninsula. The genome project is deposited in the IMG database and the complete genome sequence in GenBank (CP001643). Sequencing, finishing and annotation were performed by the GenePool Team at NERC Biomolecular Analysis Facility (NBAF) Edinburgh. A summary of the project information is shown in Table 2.

**Table 2:** Project information

**MIGS ID**	**Property**	**Term**
MIGS-31	Finishing quality	Draft
MIGS-28	Libraries used	Illumina
MIGS-29	Sequencing platforms	Illumina SOLEXA GAIIx
MIGS-31.2	Fold coverage	131×
MIGS-30	Assemblers	Velvet
MIGS-32	Gene calling method	RAST
	Genome Database release	181
	Genbank ID	866909
	Genbank Date of Release	December 12, 2010
	GOLD ID	Gi07317

## Genomic properties

The genome was assembled into 335 contigs and includes two circular chromosomes combining to give a total size of 5,353,266 bp (44.84% GC content). A total of 4,950 genes were predicted, 4,864 of which are protein-coding genes. 74.22% of protein coding genes were assigned to a putative function with the remaining annotated as hypothetical proteins. 658 protein coding genes belong to paralogous families in this genome corresponding to a gene content redundancy of 13.29%. The properties and the statistics of the genome are summarized in Tables 3-5.

**Table 3:** Summary of genome*

**Label**	**Size (Mb)**
Chromosome 1	3.4
Chromosome 2	1.9

* Two chromosomes with no plasmids. Approximate chromosome size estimated by Pulse field gel electrophoresis

**Table 5:** Number of genes associated with the 25 general COG functional categories

**Code**	**Value**	**%age**	**Description**
J	200	4.86	Translation
A	1	0.02	RNA processing and modification
K	369	8.96	Transcription
L	154	3.74	Replication, recombination and repair
B	1	0.02	Chromatin structure and dynamics
D	37	0.9	Cell cycle control, mitosis and chromosome partitioning
Y			Nuclear structure
V	75	1.82	Defense mechanisms
T	432	8.31	Signal transduction mechanisms
M	227	5.51	Cell wall/membrane biogenesis
N	148	3.59	Cell motility
U	146	3.55	Intracellular trafficking and secretion
O	173	4.2	Posttranslational modification, protein turnover, chaperones
C	203	4.93	Energy production and conversion
G	248	6.02	Carbohydrate transport and metabolism
E	348	8.45	Amino acid transport and metabolism
F	105	2.55	Nucleotide transport and metabolism
H	159	3.86	Coenzyme transport and metabolism
I	119	2.89	Lipid transport and metabolism
P	188	4.57	Inorganic ion transport and metabolism
Q	77	1.77	Secondary metabolites biosynthesis, transport and catabolism
R	445	10.81	General function prediction only
S	356	8.65	Function unknown
-	1276	25.78	Not in COGs

a) The total is based on the total number of protein coding genes in the annotated genome.

**Table 4:** Nucleotide content and gene count levels of the genome

**Attribute**	**Value**	**% of total^a^**
Size (bp)	5,353,266	100%
G+C content (bp)	2,400,750	44.87%
Coding region (bp)	4,627,782	86.45%
Total genes^b^	4950	100%
RNA genes	86	1.74%
Protein-coding genes	4864	98.26%
Genes in paralog clusters	658	13.29%
Genes assigned to COGs	3674	74.22%
Genes with signal peptides	1655	33.43%
Genes with transmembrane helices	1167	23.58%
Paralogous groups	658	13.29%

a)The total is based on either the size of the genome in base pairs or the total number of protein coding genes in the annotated genome.

b)Also includes 54 pseudogenes and 5 other genes.

## Genomic comparison

**Figure 1:**
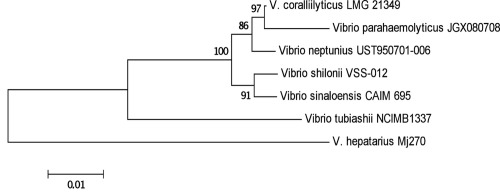
Phylogenetic tree highlighting the position of *V. tubiashii* NCIMB 1337 relative to other Vibrio strains. The tree was inferred from 1,159 aligned characters of the 16S rRNA gene sequence under the neighborhood joining criterion. Numbers above the branches are support values from 1,000 bootstrap replicates if greater than 60%.

Based on COG I.D the *Vibrio tubiashii* genome shows most similarity to the genome of *V coralliilyticus* (R^2^ = 0.96) and to *V. orientalis* (R^2^ = 0.94), while showing less similarity to *V. shilonii* (R^2^= 0.86) [Table 6]. This is in contrast to the 16S-based analysis shown in Figure 1. However, it should be noted that 16S rRNA analysis often poorly discriminates vibrios due to low sequence heterogeneity in the 16S gene [28].

**Table 6:** Comparison of the genome of *Vibrio tubiashii* NCIMB 1337 with other sequenced Vibrios

Genome Name	*Vibrio coralliilyticus* ATCC BAA-450	*Vibrio orientalis* CIP 102891	*Vibrio shilonii* AK1	*Vibrio tubiashii* NCIMB 1337
Genes	5,144	4,297	5,438	4,950
RNA	122	128	78	86
w/ Func Pred	3,687	3185	3,517	4,062
w/ Func Pred %	71.68%	74.12%	64.67%	82.06%
Enzymes	1,143	1,058	1,258	1,116
Enzymes %	22.22%	24.62%	23.13%	22.55%
KEGG	1397	1,257	1,511	1,354
KEGG %	27.16%	29.25%	27.79%	27.35%
COG	3815	3,302	4,093	3,674
COG %	74.16%	76.84%	75.27%	74.22%
Pfam	4127	3,520	4,379	3,976
Pfam %	80.23%	81.92%	80.53%	80.32%
TIGRfam	1,643	1,515	1,708	1,651
TIGRfam %	31.94%	35.26%	31.41%	33.35%
Signal peptide	1,733	1,408	1,214	1,655
Signal peptide %	33.69%	32.77%	22.32%	33.43%
TransMb	1,227	1,018	1,326	1,167
TransMb Perc	23.85%	23.69%	24.38%	23.58%
Pfam Clusters	2,183	2,091	2,163	2,186
COG Clusters	2,030	1,943	2,087	2,041
TIGRfam Clusters	1,310	1,246	1,300	1,323
GC Perc	0.46	0.45	0.44	0.45
Bases	5,680,628	4698244	5,701,826	5,353,266

### Regulatory systems

The *Vibrio tubiashii* NCIMB 1337 genome contains multiple quorum sensing systems, most notably a luxM/N system which has two adjacent copies of the luxN gene. In addition, there is a luxS/PQ system, with the lux P and Q gene appearing consecutively. There is also a cqsA/S system. It is probable that these three systems converge on the phospho-relay transfer system encoded by the luxO/luxU/hapR genes. There are two additional lux genes (LuxT and LuxZ). The genome also contains the rpoN gene encoding for the sigma-54 factor, which may indicate the presence of the two-component phosphorylation-dephosphorylation cascade described in *V. harveyi* [29] (note: *Vibrio harveyi* is also known as *Lucibacterium harveyi* and *Beneckea harveyi*.)

### Antibiotic resistance

There are six separate genes encoding for putative β-lactamases within the genome, but only two have homology at the protein levels with any know *Vibrio* β-lactamases. There is also a multi-antibiotic resistance protein MarC, associated with an operon containing a variety of multidrug resistance proteins. This operon is controlled by a MerR type transcriptional regulator, which is often associated with antibiotic resistance [30], and may account for the kanamycin resistance observed in this strain by the authors.
